# Validation of the Arabic version of the Perinatal Anxiety Screening Scale (PASS) among antenatal and postnatal women

**DOI:** 10.1186/s12884-020-03451-4

**Published:** 2020-12-04

**Authors:** Hoda Jradi, Thikrayat Alfarhan, Anas Alsuraimi

**Affiliations:** 1 King Abdullah International Medical Research Center, Ministry of National Guard Health Affairs, Riyadh, Saudi Arabia; 2 College of Public Health and Health Informatics, King Saud Bin Abdulaziz University for Health Sciences, Mail code 2350, 11426 Riyadh, Saudi Arabia; 3 College of Medicine, Alfaisal University, Riyadh, Saudi Arabia

**Keywords:** Anxiety, PASS, Pregnancy, Perinatal, Antenatal, postnatal, Arabic, Saudi Arabia

## Abstract

**Background:**

Anxiety among women in the perinatal period is common. Assessing the severity of perinatal anxiety will help monitor the progress of the patient through the stages of anxiety and facilitated the treatment. This study assesses the validity and reliability of the “Perinatal Anxiety Screening Scale” (PASS) in the Arabic language.

**Methods:**

The PASS was translated into Arabic. Two hundred seventeen women in the antenatal and postnatal phase participated (92 antenatal and 125 postnatal) answered to PASS, GHQ12, EPDS-10, and DASS-21. Content validity, factor analysis, internal consistency, and test retest reliability were assessed.

**Results:**

Content Validity Index (CVI) and Content Validity Ratio (CVR) were .88 and 0.79; respectively. The scale loaded on four components: acute anxiety, social anxiety, and dissociation; specific fears and trauma; general anxiety and adjustment; and perfectionism and control. Cronbach’s Alpha value for the scale was 0.78 and test retest correlation coefficient was 0.94. PASS significantly correlated with EPDS-10 (rho=0.46), GHQ-12(rho=0.58), the three components of DASS-21 (0.47, 0.50, and 0.43; respectively), and experiencing adverse life events.

**Conclusion:**

The Arabic translated version of the PASS showed reasonably adequate validity and reliability and can be used to screen for anxiety disorder among women in the perinatal phase.

## Background

Maternal health can be negatively impacted by untreated anxiety [1–3]. Anxiety can also negatively affect the behavioral development, emotional and cognitive status of the child [4], and the mother-infant relationship [5, 6]. For this reason, the detection of problematic anxiety via the use of an effective screening tool may be important in screening for risk of problematic anxiety, prevention, early intervention, and treatment in the field of perinatal mental health.

Knowing the severity of anxiety is important to guide the treatment for the patient [6]. Also, continuous monitoring of response to psychological and/or pharmacological treatment is essential to evaluate the effectiveness of the plan of care [7]. Depending on the severity of their symptoms, women can be assigned to maternity and childcare services, counseling services, psychosocial therapy, or to psychiatric hospitalization in severe cases [6].

A validated tool that assesses the severity of perinatal and postnatal anxiety will help monitor the progress of the patient through the stages of anxiety and facilitated the adjustment of the treatment approach as required [8, 9].

There are no available scales in the Arabic language to assess antenatal anxiety and classify patients according to the stages of severity of symptoms. The Depression, Anxiety and Stress Scale (DASS-21) [10] have been validated in Arabic and is used among the general population. However, this scale measures are limited in classifying the severity of anxiety in the perinatal period because it has not been validated in perinatal samples and failed to include measures of anxiety related to pregnancy [11]. The Edinburgh Postnatal Depression Scale (EPDS [12] has also been validated in Arabic and is a self-administered scales that screens for depression and classifies women at risk for anxiety if they score over a pre-established cut-off score ; however, the scale does not classify the severity of anxiety according to pre-established severity ranges. The EPDS is lacking in psychometric sensitivity for screening for anxiety disorders [12, 13].

The Perinatal Anxiety Screening Scale (PASS) is a 31-item self-administered scale to screen for anxiety in antenatal and postnatal women that was developed and validated among a sample of pregnant women in 2014[14]. The scale includes measures such as fear of birth or fear that harm will come to the baby. It has a four-factor structure that assesses symptoms of acute anxiety, worry and fear, perfectionism, control and trauma, and social anxiety [14]. The validity and reliability study of the original scale reported a good internal consistency for the four dimensions of the scale (coefficient alpha of 0.90, 0.89, 0.86, and 0.87; respectively) and a test re-test correlation coefficient of 0.74 [14]. The correlation between the PASS and other measures of depression and anxiety ranged between 0.77 and 0.83. A cut-off score of 26 was established to identify 68% of women at risk of an anxiety disorder, severe anxiety as 42 to 93, mild to moderate anxiety as 21–41, and minimal anxiety as 0 to 20 [14, 15]. Additionally, the Turkish version of the scale (PASS-TR), showed high sensitivity and specificity for a self-report scale, a cut-off value of 16, a high total internal consistency (coefficient alpha of 0.95), and correlated positively with other existing valid and reliable measures of anxiety [16].

The main aim of this study is to validate the Perinatal Anxiety Scale (PASS) in the Arabic language to increase its usefulness as a screening tool for identifying and tracking problematic antenatal and postnatal anxiety.

## Methods

### Participants recruitment and data collection

This study was used a cross-sectional methodological design. A sample of 217 perinatal (92 in antenatal phase and 125 in postnatal phase) were enrolled in the validity and reliability study of the Arabic version of the PASS scale (7 participants :1survey item). Sample size was judged sufficient according to the recommendations of a minimum subject to item ratio of at least 5:1 [17]. Women were recruited randomly as they entered the clinics from the obstetrics/gynecology and pediatric clinics at King Fahad National Guard Hospital in Riyadh. The chosen clinics provide care for women and their infants from different socioeconomic backgrounds. The clinic serves personnel from the military, their families, in addition to employees affiliated with the hospital and a large university system and their dependents. Surveys were collected anonymously and it took approximately 15 minutes for completion. Illiterate women and those with a disability that prevents them from participating in the study were excluded. Written consent was obtained from all participants. Data collection continued for a period of three months.

### Translation and validation process

The validation process started with the standard “forward-backward” procedure [18] by translating the original version of the PASS questionnaire from English into classical Arabic by two independent professional translators and subsequently back translated into English by Two other independent translators and checked for consistency. Content validity of the translated Arabic version was assessed by presenting the translated instrument to a group of experts (an obstetrics/gynecology physician, two psychologists, and two nurses and a health educator from the maternity clinic). The group evaluated the relevance and cultural appropriateness of each item of the scale. Content Validity Ratio (CVR) and Content Validity Index (CVI) were generated. the Arabic version and the English version of the tool were administered consecutively within 15 min to a group 15 bilingual female office employee. Correlation of the English and Arabic version was calculated using the Spearman correlation coefficient (rho). Finally, the translated Arabic version of the PASS questionnaire was piloted in a sample of new mothers (*N* = 20) to ensure clarity of all terms. Additionally, the convergent validity of the PASS was assessed by concurrently administering the Arabic versions of the Edinburgh Postpartum Depression Scale (EPDS-10) and the General Health Questionnaire (GHQ-12) and the Depression, anxiety, and Stress Scale 21 (DASS-21) to study participants. Both the EPDS-10 and the GHQ-12 are valid and reliable scales in the Arabic language previously used to screen for risk of depression and risk of psychological distress in the prenatal and postpartum period [19, 20]. The DASS-21 is also a valid and reliable scale in the Arabic language that consists of 21 items measuring depression, anxiety, and stress with higher scores indicating higher level of psychological distress in all three domains [21].

Participants were also asked to complete a demographic questionnaire (age, gender, marital status, employment status, level of education, and income), and questions related to adverse life events experienced within the last year (divorce in the family, problems at work or university, death in the family, illness in the family, financial problems, other personal problems, or health problems related to pregnancy or delivery). We anticipated that a positive correlation between these negative life events and the PASS will add to the validity of the scale.

### Statistical analysis

Responses were collected anonymously and once the data collection process was completed, data were imported from the excel sheets into Stata (version 14.0, College Station, TX: StataCorp LLC), for analysis purposes. All scales in this study were scored according to recommendations, and means and standard deviations were calculated for each scale. Composite variables were generated based on the final scores as high, moderate, or low to indicate levels of psychological distress among participants for all scales. Study variables were summarized, in aggregate, using standard descriptive statistics and frequencies of responses were reported. Content validity Ratio and Content Validity Index were calculated for content validity check. Factor structure of the questionnaire was performed using principal component analysis (PCA) with Varimax rotation. The reliability of the scale was checked via internal consistency and test retest reliability. Cronbach’s alpha coefficient was reported for internal consistency (alpha ≥ 0.70 was considered acceptable) ([22]. Test-retest reliability was conducted (*N* = 30) within one week interval and a correlation coefficient was obtained.

## Results

### Participants’ characteristics

Overall, 283 women were recruited for this study. Twenty of them were consented for the initial piloting phase, 30 of them for the test re-test reliability, 16 withdrew before completing the survey and were not included in the final sample size; the remaining sample of 217 women were included in the final sample size for this study. 

The mean age of participants was 28.89 years (range between 19 and 44) with a standard deviation of 7.07 years. Most of the sample (70.51%) reported having a college degree. The majority (58.06%) did not work and 56.28% reported that their income was adequate for their lifestyle; an income of less than 10,000 Saudi Riyals (SAR) was reported by approximately half (47.69%) of the sample. Using the suggested cut-off values for the English version of the PASS scale [14] of 0–20 (risk of minimal anxiety), 21–41 (risk of moderate anxiety), and above 41 (risk of severe anxiety), 34.10% of the participating women reported a risk of moderate anxiety and 47.0% reported a risk of severe anxiety. Participants’ characteristics are presented in Table 1.

**Table 1:** Characteristics of study participants (*N*=217)

Characteristics	N	%
**Age (μ=28.89±7.07)**		
18-25	51	23.50
26-34	126	58.06
≥35	40	18.30
**Marital Status**		
Married	214	98.62
Divorced/Separated/Widowed	3	1.38
**Level of Education**		
Highschool or less	64	29.49
College or more	153	70.51
**Employment Status**		
Unemployed	127	58.52
Employed/Student	90	41.47
**Income (SR)**^**a**^		
< 10,000	104	47.46
≥10,000	113	52.07
**Pregnancy Stage**		
Antenatal	92	42.40
Postnatal	125	5760
**Level of Anxiety (PASS)**		
Low	41	18.89
Moderate	74	34.10
High	102	47.0

^**a**^***SR*Saudi Riyals**

Frequency of responses to each of the questions of the Arabic version of the PASS scale are displayed in Table 2. The mean average score for the Arabic version of the PASS scale was 41.01(SD = 20.55) (range: 0 to 93). The mean EPDS-10 score was 17.57 (SD = 4.32) (range: from 0 to 30) with 82.40% of the surveyed women scoring above 13 for depression symptoms (indicative of risk of depression) [12]. Additionally, the mean score for GHQ-12 was 10.81(SD = 7.30) (range: 0 to 36) and 36.87% of participants exhibited an above 12 score (indicative of risk mental disorder and risk of psychological distress) [23]. The mean scores for the three components of the DASS-21 were 11.04 (SD = 9.70) for depression, 11.73 (SD = 9.46) for anxiety, and 17.10 (SD = 10.26) for stress symptoms. Almost 37% of the participants reported a risk of severe and extremely severe anxiety according to the DASS-21. Mean scores for the four scales and the composite score for adverse life events are presented in Table 3.

**Table 2:** Participants’ response to the Arabic version of the PASS scale (*N*=217)

PASS Items	Not at all N (%)	Sometimes N (%)	Often N (%)	Almost Always N (%)
**Total anxiety score (μ=41.02; SD=20.55)**				
1.Worry about baby/pregnancy	69 (31.80)	79 (36.41)	46 (21.20)	23 (10.63)
2.Fear that harm will come to the baby	78 (35.94)	78 (35.94)	40 (18.43)	21 (9.68)
3.A sense of dread that something will come to the baby	56 (25.81)	65 (29.91)	32 (14.75)	64 (29.49)
4.Worry about many things	55 (25.35)	88 (40.55)	41 (18.89)	33 (15.21)
5.Worry about the future	56 (25.81)	62 (28.57)	27 (12.44)	72 (33.18)
6.Feeling overwhelmed	35 (15.67)	5 (23.50)	33 (15.21)	99 (45.62)
7.Really strong fears about things (needles, blood, birth, pain)	45 (20.74)	72 (33.18)	53 (24.2)	47 (21.66)
8.Sudden rush of extreme fear or discomfort	30 (13.82)	59 (27.19)	46 (21.20)	82 (37.79)
9.Repetitive thoughts that are difficult to stop or control	44 (20.28)	70 (32.26)	38 (17.51)	65 (29.95)
10.Difficulty sleeping even when I have the chance to sleep	33 (15.21)	72 (33.18)	42 (19.35)	70 (32.26)
11. Having to do things in a certain way or order	35 (16.93)	78 (35.94)	52 (23.96)	52 (23.96)
12.Wanting things to be perfect	68 (31.34)	53 (24.24)	51 (23.50)	45 (20.74)
13.Needing to be in control of things	57(26.27)	65 (29.95)	50 (23.04)	45 (20.74)
14.Difficulty stopping checking things over and over	42 (19.35)	73 (33.64)	36 (16.39)	66 (30.41)
15.Feeling jumpy or easily startled	33 15.21)	58 (26.73)	42 (19.35)	84 (38.71)
16.Concerns about repeated thoughts	39 (17.97)	77 (35.48)	38 (17.51)	63 (29.0)
17. Being on guard or needing to do watch out for things	60 (27.65)	89 (41.01)	32 (14.75)	36 (16.59)
18.Upset about repeated memories, dreams, or nightmares	40 (18.43)	61 (28.11)	30 (13.82)	86 (39.63)
19.Worry that I will embarrass myself in front of others	34 (15.67)	58 (26.73)	34 (15.67)	91 (41.94)
20.Fear that others will judge me negatively	36 (16.59)	58 (26.73)	28 (12.90)	95 (43.78)
21.Feeling really uneasy in crowds	31 (14.29)	46 (21.20)	29 (13.36)	111 (51.15)
22.Avoiding social activities because I might be nervous	26 (11.98)	43(19.82)	27 (12.44)	121 (55.76)
23. Avoiding things which concern me	53 (24.24)	59 (27.19)	44 (20.28)	61 (28.11)
24.Feeling detached like watching yourself in a movie	28 (12.90)	32 (14.75)	14 (6.45)	143 (65.90)
25.Loosing track of time and can’t remember what happened	31 (14.29)	54 (24.88)	25 (11.52)	107 (49.31)
26.Difficulty adjusting for recent changes	34 (15.67)	49 (22.58)	33 (15.21)	101 (46.54)
27.Anxiety getting in the way of being able to do things	32 (14.75)	79 (36.41)	37 (17.05)	69 (31.80)
28.Racing thoughts making it hard to concentrate	33 (15.21)	68 (1.34)	43 (19.82)	73 (33.64)
29.Fear of losing control	36 (16.59)	59 (27.19)	34 (15.67)	88 (40.55)
30.Feeling panicky	26 (11.98)	45 (20.74)	26 (11.98)	120 (55.30)
31.Feeling agitated	25 (11.52)	37 (17.05)	27 (12.44)	128 (58.99)

**Table 3:** Distributions of scales (medians, means, and standard deviations) and correlations with PASS

Scale	Median	Mean (SD)	Range	Spearman’s Rho (***p***-value)
PASS	39	41.02(20.55)	3-97	-
EPDS-10	18	17.57 (4.32)	5-26	0.46 (<0.001)
GHQ-12	9	10.8 (7.30)	0-33	0.58 (<0.001)
DASS-21 (depression)	8	11.04 (9.70)	0-42	0.47 (<0.001)
DASS-21 (anxiety)	10	11.73 (9.46)	0-40	0.50 (<0.001)
DASS-21 (stress)	16	17.10 (10.26)	0-42	0.43 (<0.001)
Adverse life events (total)	6	6.7(5.4)	0-19	0.28 (0.003)
Divorce	0	0.54 (108)	0-4	0.11 (0.10)
Problems at work	0	0.64 (0.97)	0-3	0.2 (0.002)
Death in the family	0	0.96 (1.31)	0-3	0.06 (0.42)
Illness in the family	0	0.97 (1.27)	0-3	0.16 (0.02)
Money problems	1	1.16 (1.11)	0-3	0.18 (0.007)
Personal problems	1	1.11 (1.12)	0-3	0.23 (0.0005)
Pregnancy problems	1	1.32 (1.17)	0=3	0.12 (0.07)

### Reliability of the instrument

Internal consistency using Cronbach’s alpha coefficient as a measure of reliability of the Arabic version of the PASS questionnaire was found to be 0.94 for the whole sample indicating very satisfactory results. It was 0.83, 0.80, 0.86, and 0.90 for the four sections of the scale. The results of the test-retest reliability of the Arabic version of the PASS tool showed to be acceptable with a correlation coefficient of 0.78.

### Validity of the instrument

Validity of the instrument was assessed using convergent validity. The correlations between the Arabic version of PASS, the Arabic version of the EPDS-10, the Arabic version of the GHQ-12, the three components of the DASS-21 (Depression, Anxiety, and Stress), and the score of the stressful life events within the last 12 months were significantly positive (Spearman’s rho = 0.46; *p *< 0.001, Spearman’s rho = 0.58; *p* < 0.001, Spearman’s rho = 0.47; *p* < 0.00’ Spearman’s rho = 0.50;*p* < 0001, spearman’s rh = 0.43; *p* < 0.001, and Spearman’s rho = 0.28; *p* = 0.003; respectively), indicating that those who had perinatal anxiety showed higher levels of depression and higher levels of psychological disorders. Particularly those who reported experiencing problems at work, illness in the family, money problems, and personal problems significantly correlated with the PASS scores (Table 3).

Results of the content validity results from the expert panel displayed a mean Content Validity Ratio (CVR) of 0.79 and a mean Content Validity Index (CVI) of 0.88.

### Factor structure

In order to confirm the structure of the Arabic version of the instrument and demonstrate its construct validity and similarity to the English version, the Principal Component Method for factor analysis with varimax rotation was applied using the original four factor structure of the instrument. The four factors jointly accounted for 54.53% of the total detected variance. Factor 1 (acute anxiety, social anxiety, and dissociation) accounts for 37.12% of the total variance, Factor 2 (specific fears and trauma) 7.51%, Factor 3 (general anxiety and adjustment) 5.20%, and factor 4 (perfectionism and control) 4.11%. Results for factor structure of the scale are presented in Table 4.

**Table 4:** Results of factor analysis: factor structure and factor loadings

PASS Items	Factor 1	Factor 2	Factor 3	Factor 4
**Factor 1: Acute anxiety, Social Anxiety, and Dissociation**				
8.Sudden rush of extreme fear or discomfort	0.55			
15.Feeling jumpy or easily startled	0.64			
19.Worry that I will embarrass myself in front of others	0.64			
20.Fear that others will judge me negatively	0.55			
21.Feeling really uneasy in crowds	0.73			
22.Avoiding social activities because I might be nervous	0.65			
24.Feeling detached like watching yourself in a movie	0.70			
25.Loosing track of time and can’t remember what happened	0.59			
23. Avoiding things which concern me	0.70			
28.Racing thoughts making it hard to concentrate	0.51			
29.Fear of losing control	0.62			
30.Feeling panicky	0.78			
31.Feeling agitated	0.82			
**Factor 2: Specific Fears, and trauma**				
1.Worry about baby/pregnancy		0.59		
2.Fear that harm will come to the baby		0.72		
3.A sense of dread that something will come to the baby		0.62		
4.Worry about many things		0.68		
5.Worry about the future		0.40		
7.Really strong fears about things (needles, blood, birth, pain)		0.44		
9.Repetitive thoughts that are difficult to stop or control		0.54		
10.Difficulty sleeping even when I have the chance to sleep		0.53		
16.Concerns about repeated thoughts		0.52		
18.Upset about repeated memories, dreams, or nightmares		0.49		
**Factor 3: General Anxiety and Adjustment**				
6.Feeling overwhelmed			0.47	
17. Being on guard or needing to watch out for things			0.62	
26.Difficulty adjusting for recent changes			0.72	
27.Anxiety getting in the way of being able to do things			0.51	
**Factor 4: Perfectionism and Control**				
11. Having to do things in a certain way or order				0.44
12.Wanting things to be perfect				0.76
13.Needing to be control of things				0.66
14.Difficulty stopping checking things over and over				0.57
**Variance (%) (total: 54.53%)**	37.12	7.51	5.20	4.11

## Discussion

This is the first study conducted to evaluate the validity and reliability of the Arabic Perinatal Anxiety Scale. This study showed that the Arabic version of the PASS is valid and reliable for use in the Arabic speaking populations and have good psychometric properties. Women who participated in this validation study were in the antenatal and postnatal phase, reflecting the suitability of the scale for both phases. Also, participants were from different age groups and different socioeconomic status.

The reliability coefficient was assessed and the Arabic version of PASS, similarly to the original English version of the scale (0.95) [14] and the Turkish version of the scale (0.96) [16], demonstrated high reliability (0.94). The test retest reliability of 0.78 was acceptable and comparable to the test retest reliability of the original scale of 0.74 [14].

The factor analysis was conducted with retaining the four components structure of the original version of the scale. Similarly, to the original English version [14] and the Turkish version [16] of the PASS, the questions loaded on the four factors; however, the actual distribution of the questions for the Arabic version of the PASS varied across the four components. The original scale factor structure were “acute anxiety and adjustment” for factor 1 (constructs related to panic disorder, dissociative disorder, and adjustment difficulties), “general worry and specific fear” for factor 2 (constructs related general anxiety disorder and phobia), “perfectionism, control and trauma” for factor 3 (constructs related to obsessive compulsive disorder and posttraumatic stress disorder), and “social anxiety” for factor 4 (constructs related to social anxiety) [14]. For the Arabic PASS, factor 1 included acute anxiety, social anxiety, and dissociation; factor 2 included specific fears and trauma; factor 3 included general anxiety and adjustments; and factor 4 included perfectionism and control. Actually, these results of factor analysis are similar to those obtained for the Turkish version of the scale on factor 4 (perfectionism and control) and partially for the other three factors [16]. These changes in the structure of components distribution for the scale may reflect the cultural and linguistic adaptation of the scale to the Arabic speaking population.

As hypothesized, the Arabic PASS had positive significant associations with previously validated Arabic versions of the EPDS-10, GHQ-12, and DASS-21. It is not surprising that the PASS correlated positively with the EPDS since anxiety and depression are known to occur concurrently in more than 50% of the time [24]. Results are indicative that some of the women in this group may suffer from anxiety with or without depression. The PASS can identify anxiety status regardless of the depression status among women in the antenatal and postnatal stage. Further studies are recommended for assessing the ability of the PASS to distinguish between those who have anxiety only or in combination with other psychological disorders among pregnant and postpartum women. There was also a positive, significant, but weaker, association between the PASS and the adverse life events. This existing low magnitude association may be due to the way people cope with adverse life events [25]. For example, persons with good coping strategies may score low on the Arabic PASS even though their score for adverse life events is high. It is highly recommended to conduct further investigation regarding experienced life events and anxiety and the impact of coping strategies.

The PASS scale is composed of 31 items which is considered too long for a screening test; particularly in the clinical setting. However, it is useful for researchers in all settings because of its capacity of detecting anxiety disorders at higher rates than other scales and including broad constructs of anxiety disorder not present in other scales. Levels of anxiety (47%) detected by the Arabic version of the scale among this sample were not far from the values detected by the Turkish version (50%) for their recruited samples [16]. The mean scores reported for the Turkish version seem to be lower because they used a modified scoring techniques and different cut-off value from the original scale to adapt it to cultural differences in responding [16]. The Arabic version of the PASS was validated against the EPDS-10, GHQ-12, DASS-21, and constructs associated with adverse life events and not against a standard diagnostic instrument or criteria use in the clinical setting, making it of limited use in the clinical setting and with no recommendations for a cut-off values that can be used for diagnostic purpose. Further validation work on establishing clinical cut off scores and severity ranges for the PASS in Arabic populations is needed.

This study has some limitations. Participants were recruited from clinics in the Eastern part of Riyadh and the sample cannot be considered representative of the entire population of Riyadh or the Saudi population. However, formal Arabic was used to translate and validate the tool, making it comprehensible and useful for all Arabic speaking nations.

## Conclusions

This study provides the literature with an Arabic scale for assessing anxiety during the antenatal and postnatal period. The Arabic version of the PASS showed to be valid and reliable and can be used as a screening tool for anxiety among the Arabic speaking population.

## Data Availability

The datasets analysed during the study are available from the corresponding author on reasonable request and after approval of the concerned authorities in the organization.

## Abbreviations

PASS: Perinatal Anxiety Screening Scale
EPDS: Edinburgh Postnatal Depression Scale
DASS: Depression, Anxiety, and Stress Scale
GHQ: General Health Questionnaire
CVR: Content Validity Ratio
CVI: Content Validity Index
SAR: Saudi Riyals
